# The quality of communication about older patients between hospital physicians and general practitioners: a panel study assessment

**DOI:** 10.1186/1472-6963-7-133

**Published:** 2007-08-24

**Authors:** Helge Garåsen, Roar Johnsen

**Affiliations:** 1 Department of Public Health and General Practice, Faculty of Medicine, The Norwegian University of Science and Technology (NTNU), Trondheim, Norway

## Abstract

**Background:**

Optimal care of patients is dependent on good professional interaction between general practitioners and general hospital physicians. In Norway this is mainly based upon referral and discharge letters. The main objectives of this study were to assess the quality of the written communication between physicians and to estimate the number of patients that could have been treated at primary care level instead of at a general hospital.

**Methods:**

This study comprised referral and discharge letters for 100 patients above 75 years of age admitted to orthopaedic, pulmonary and cardiological departments at the city general hospital in Trondheim, Norway. The assessments were done using a Delphi technique with two expert panels, each with one general hospital specialist, one general practitioner and one public health nurse using a standardised evaluation protocol with a visual analogue scale (VAS). The panels assessed the quality of the description of the patient's actual medical condition, former medical history, signs, medication, Activity of Daily Living (ADL), social network, need of home care and the benefit of general hospital care.

**Results:**

While information in the referral letters on actual medical situation, medical history, symptoms, signs and medications was assessed to be of high quality in 84%, 39%, 56%, 56% and 39%, respectively, the corresponding information assessed to be of high quality in discharge letters was for actual medical situation 96%, medical history 92%, symptoms 60%, signs 55% and medications 82%. Only half of the discharge letters had satisfactory information on ADL. Some two-thirds of the patients were assessed to have had large health benefits from the general hospital care in question. One of six patients could have been treated without a general hospital admission. The specialists assessed that 77% of the patients had had a large benefit from the general hospital care; however, the general practitioners assessment was only 59%. One of four of the discharge letters did not describe who was responsible for follow-up care.

**Conclusion:**

In this study from one general hospital both referral and discharge letters were missing vital medical information, and referral letters to such an extent that it might represent a health hazard for older patients. There was also low consensus between health professionals at primary and secondary level of what was high benefit of care for older patients at a general hospital.

## Background

The effectiveness and quality of care for older patients is largely dependent on the content of the written communication between physicians; i.e. referral and discharge letters. There is consensus between clinicians on the content of the referral [1] and discharge letters [2].

Still, national and international studies show an insufficient quality in the written communication about patients' medical situation and in the transferral of duties and obligations from one responsible person or medical team to another [3-16]. Studies have shown that initial short reports [12,13], joint charts [14], electronic interactive referrals [15] or structured communication formulas [16] have not, or have only partly, improved the quality of communication between physicians.

Fatal adverse drug events have become a major hospital problem, especially for older patients with multiple diseases and a high number of administrated drugs [6,17,18].

Health care provision in Norway is based on a decentralised model. The municipalities (primary health care) are responsible for home care services, nursing homes, community hospitals, family physicians, health services for mothers, children and youth, midwives, physiotherapists, occupational therapists and emergency services provided by general practitioners on duty. The government (secondary health care) owns and runs district general hospitals, university hospitals and ambulance services throughout the regional health authorities (five regions). Professional collaboration between physicians in primary health care and secondary health care is mainly based on written communication in the form of referral and discharge letters. Direct contact, by telephone or in meetings, occurs only in special incidents.

Since 2002 one of the official Norwegian health quality criteria is the quality of the discharge letters. However, the quality of the discharge letters in Norway in 2007 still remains modest [19].

The main objectives of the present study were to assess the quality of written communication about older patients between physicians and to estimate the number of patients that could have been treated at primary level instead of at a general hospital by using a Delphi technique with two expert panels comprising hospital physicians, general practitioners and public health nurses.

## Methods

### Setting

During a three week period in February 2002 100 referral and discharge letters, both acute and elective, were included consecutively. The city general hospital in Trondheim, St.Olavs University Hospital, is both a general hospital for the municipality of Trondheim and a university hospital for the three counties in Mid-Norway. In this study only patients being admitted to the general hospital were included.

The study population was patients 75 years of age or older admitted to the orthopaedic (n = 30), pulmonary (n = 30) and cardiological (n = 40) departments from the municipalities of Trondheim and Malvik. There were no exclusion criteria. Secretaries at the general hospital collected copies of all referral and discharge letters for the included patients when discharge letters were signed. Neither the general practitioners nor the general hospital physicians knew which patients were included in the study, as the time for inclusion was unknown to the physicians.

### Study design

Two expert panels were recruited. Each panel consisted of one general hospital physician (geriatrician), one general practitioner and one public health nurse. All of the panel members were certified specialists in their respective fields. None of them had any affiliation with the departments involved in the study.

The panels used a standardised evaluation protocol with a visual analogue scale (VAS). The panels assessed the quality of the written information concerning the patients' actual situation, former medical history, symptoms, signs, medication, social network, activity of daily living (ADL), need of care and responsibility for follow-up care. The information was judged as to whether it was sufficient or not according to the patients presented problems or diagnoses. The panels also assessed the level of benefit gained by general hospital care and if the patients could be treated outside the general hospital; at a nursing home, community hospital, a rehabilitation department, an outpatient department at the general hospital, by public home care services or by a general practitioner. The aim was to estimate the number of patients that could have been treated without admission to the general hospital.

Before this study began, a pilot study of five referral letters was carried out where the expert panels examined, discussed and tested the evaluation protocol thoroughly in two meetings.

In the study twenty-five referral and discharge letters were evaluated by both panels; 15 from cardiological, five from pulmonary and five from orthopaedic departments. The rest of the referral and discharge letters were assessed by only one of the expert panels.

Each panel member examined copies of the referral and discharge letters individually. Consensus was defined to exist only if the difference between the group members did not exceed two on the VAS scale. If this criterion was met, the panel's evaluation was defined as the median of the three group members. Otherwise, the case was discussed in a meeting, using the Delphi technique [20], with all the participants of the panel. This methodology was also used for cases evaluated by both panels. To show the level of consensus between the panels the 25 referral and discharge letters evaluated by both panels are presented separately. The panels' assessments, as well as each expert's, were recorded for each referral and discharge letter.

All data was blinded with respect of the patients' identity (name, birthday and address), the name of the departments at the general hospital and the names of the physicians.

The Regional Committee for Medical Research Ethics for Central Norway approved the study. The study was granted license by the Norwegian Data Inspectorate and all data was processed in anonymous form.

### Statistical methods

To investigate the structure of the consensus between the participants in each panel and between the panels it was decided, during the assessments in the pilot study, to divide the assessments into three categories; low (1–3), intermediate (4,5) and high (6–8), and the results were tabulated against each other in contingency tables.

We undertook all analysis using SPSS version 14.0 for Windows and Excel version 2003. Differences between the departments were tested by chi square tests. Statistical significance was set at p = 0.05.

**Figure 1:**
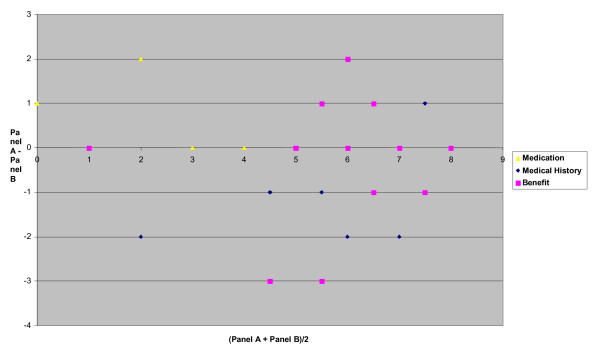
The difference between the mean score of the quality of information on medication, medical history and the benefit of general hospital care from Panel A and Panel B according to the assessed quality. Bland and Altman diagram.

Data was collected, on all assessments of the 25 cases assessed by both panels, for interrater and test reliability analyses. Agreement between the panels and within each panel was estimated as observed and proportional agreement together with kappa statistics [21,22]. Strength of consensus (value of κ) was defined as: very good (0.81 – 1.00), good (0.61 – 0.80), moderate (0.41 – 0.60), fair (0.21 – 0.40) and poor (below 0.20). The distribution of concordance was also analysed with a Bland-Altman diagram (Figure 1) [23].

## Results

### Referral letters

None of the patients were referred from the same physician. The patients' usual general practitioner referred 19 patients, 44 by general practitioners on emergency care duty, 23 by ambulance personnel, and for 14 patients the signature of the referring physician was unreadable. The description of the actual medical situation leading to the referral was denoted to be of high quality in 84%, of former medical history in 39%, of symptoms in 56%, of signs in 56% and of medication in 39% of the cases (Table 1). Descriptions of the patients' social network and need for home care were assessed to be of low quality in 92% and 88% of the referral letters.

**Table 1:** Assessments (with 95% Confidence Intervals) of the quality of the referral letters (N = 100)

	Low	Intermediate	High	Mean score
Actual situation	14 (8–22)	2	84 (75–91)	6,90 (6.65–7.14)
Former medical history	44 (34–54)	17	39 (29–49)	4.67 (4.17–5.17)
Symptoms	26 (18–36)	18	56 (46–66)	5.75 (5.41–6.13)
Signs	26 (18–36)	18	56 (46–66)	5.98 (5.61–6.34)
Medication	44 (34–54)	17	39 (29–49)	3.20 (2.53–3,87)
ADL	55 (45–65)	23	22 (14–-31)	3.68 (3.24–4.12)
Social network	92 (85–97)	0	8 (4–15)	1.10 (1.03–1.16)
Need of care	88 (80–94)	0	12 (6–20)	1.14 (1.07–1,22)

The quality of the referral letters were assessed to be insufficient independent of who referred the patients; general practitioners, emergency personals or physicians at outpatient departments.

### Discharge letters

The discharge letters were written by 94 different physicians. Information about the actual medical situation was assessed to be of high quality in 96%, of medical history in 92%, of symptoms in 60%, of signs in 55%, of medication in 82% and of ADL in 50% of the discharge letters (Table 2). However, the descriptions of social network (20%) and the need for home care (31%) were denoted to be of high quality in fewer cases (Table 2).

**Table 2:** Assessments (with 95% Confidence Intervals) of the quality of the discharge letters (N = 100)

	Low	Intermediate	High	Mean score
Actual situation	1 (0–5)	3	96 (90–99)	7.29 (7.10–7.48)
Former medical history	5 (2–11)	3	92 (85–97)	6.84 (6.56–7.12)
Symptoms	28 (24–32)	12	60 (51–69)	5.30 (4.86–5.74)
Signs	31 (22–41)	14	55 (45–65)	5.14 (4.70–5.58)
Medication	12 (7–20)	6	82 (73–89)	6.93 (6.47–7.40)
ADL	16 (9–25)	34	50 (40–60)	5.35 (5.02–5.68)
Social network	80 (71–87)	0	20 (13–29)	1.20 (1.12–1.28)
Need of care	69 (59–78)	0	31 (22–41	1.87 (1.73–2.00)

As much as 20% of discharge letters were missing vital medical information and almost none described ADL or patients' need for home care services.

### Benefit of general hospital care

The assessments showed that the specialists meant that general hospital care had a large beneficial value for 77% of the patients, nurses scored 71% and general practitioners 59%. The score for all the panellists combined was 70% (Table 3). Consensus regarding benefit of the admissions was fair between the panels, but varied from poor to good within the panels and between the professions; with a much higher degree of consensus between the specialists (κ = 0.64) than the other professions. Within the panels there was an especially large disagreement as to the benefit of general hospital care between the specialist and the general practitioner in one of the panels (B) (κ = 0.04).

**Table 3:** The assessment (with 95% Confidence Intervals) of health benefits of general hospital care by each profession and by both panels (N = 100)

	Low	Intermediate	High	Mean score
Hospital physicians	8 (4–15)	15	77 (68–85)	6.39 (6.04–6.73)
General Practitioners	15 (9–24)	26	59 (49–69)	5.74 (5.35–6.13)
Public Health Nurses	6 (2–13)	23	71 (61–80)	6.28 (5.97–6.58)
Both panels together	8 (4–15)	22	70 (60–79)	6.27 (5.96–6.58)

In the present study there were no statistically significant associations between the quality of the referral and discharge letters and the assessments of the benefit of the general hospital care, except for ADL. A good description of ADL, however, was strongly associated with a high benefit of general hospital care (p < 0.001).

### Follow-up responsibility after discharge

Some one of four discharge letters had no information as to who was responsible for follow-up care. Fifty-three of the patients were to be followed-up by general practitioners, 17 at outpatient departments at the general hospital, two at a nursing home, 28 needed public home care services and 23 discharge letters had no information about follow-up responsibility.

### Where could patients have been treated instead of being admitted to the general hospital

There was consensus within the expert panels that several patients could have been treated without a general hospital admission. Three of the patients could have received sufficient care from general practitioners, five by home care providers and eight at outpatient departments at a community hospital. More patients treated at the cardiological department (15% of the patients) could have been treated at outpatient departments than at the other departments. However, more patients from pulmonary (26.7%) and orthopaedic (23.3%) departments could have been treated at a community hospital than patients from the cardiological department (2.5%); a statistically significant difference (p = 0.001). The nurses (28 patients) and the general practitioners (18 patients) assessed that more patients could have been treated at a community hospital than the specialists (15 patients).

### Consensus between the expert panels, within panels and between the professions

The consensus between the panels, and within the panels and between the panellists, was very good when assessing information about the actual medical situation and former medical history (Table 4, Figure 1). We found very good agreement on medication (κ = 1.00) between the panels and from moderate to very good consensus between the same professions and within the panels. When assessing symptoms, signs, social network and need for home care, there was poor consensus between the panels and from poor (none) to moderate within the panels and within the same professions. Assessing ADL, we found a fair consensus between the panels and from poor (none) to good consensus within the panels and between the same professions.

**Table 4:** Assessments of consensus between panels A and B on the quality of the referral letters (n = 25)

	**Observed agreement**	**Proportional agreement**			
		**Low**	**Intermediate**	**High**	***κ *(95% CI)**

Actual situation	0.84	-	0.00	0.84	0
Former medical history	0.96	1.00	0.50	0.96	0.78 (0.37–1.00)
Symptoms	0.52	0.09	0.00	0.52	0.11 (0.00–0.53)
Signs	0.52	0.09	0.00	0.52	0.10 (0.00–0.47)
Medication	1.00	1.00	1.00	1.00	1.00 (1.00–1.00)
ADL	0.58	0.25	0.18	0.58	0.25 (0.00–0.61)
Social network	0.44	0.67	0.33	0.13	0.14 (0.00–0.44)
Need of care	0.72	0.75	0.15	0.17	0.51 (0.20–0.82)
Benefit of care	0.79	0.00	0.40	0.77	0.35 (0.00–0.86)

Also the Bland-Altman diagram showed small variations between the panels (Figure 1). On medication there were no differences between the panels for 21 persons, a difference of one in three cases and two in one case. The largest degree of disagreement between the panels was in relation to the level of benefit gained from general hospital care, with eleven cases with zero and one in difference, and one with two and two with three in difference. The disagreements between the panels occurred mainly when there was a low or medium score on the VAS-scale. Panel (B) had the highest score in nearly all of the 25 cases.

## Discussion

In this study from one general hospital we assessed the quality of both referral and discharge letters about older patients to be insufficient in an alarmingly large number of cases. The referral letters were of inappropriate quality in a majority of the cases in all of the assessed fields, except for the actual medical situation, that led to the referral. As less than 20 per cent of the patients were referred from general practitioners' consulting rooms most of the referral letters were written in out of office situations where the patient's medical records were not available to the referring physicians. This explanation was not applicable for the discharge letters. Nonetheless, many discharge letters were missing vital medical information, did not specify who was responsible for follow-up care and almost none described ADL and the need for home care services. However, the discharge letters were assessed to be of high quality in the majority of cases as far as actual medical situation and former medical history were concerned.

The credibility of a consensus technique depends heavily upon the composition of the panel. Some studies have shown that panels made up with stakeholders with different backgrounds were rating the same statements differently [24,25]. In all likelihood each profession will have difficulty formulating a definition of quality or a gold standard that will be relevant for other professions. Several studies have shown that expert panels composed of appropriate and multidisciplinary experts are able to make valid judgments [24-26]. However, in this study we used two different expert panels and the level of consensus between the panels was presented separately to minimise each stakeholder's effect on the results.

This study focused on the quality of letters between physicians about older patients. Older patients are affected more than younger patients by the consequences of their medical condition as far as their ability to cope in daily activities are concerned. Serious consequences can occur for older patients when letters between primary level and secondary level, and vice versa, have incomplete information about ADL, medication and patient's network. This is especially the case if there are uncertainties as to who is responsible for the follow-up care and as to what needs to be followed-up. Older patients, many with reduced mental capacity, are those most dependent on a health care system that is able to communicate appropriately and to transfer information and duties properly.

The general hospital physicians in the panels had a higher confidence in the benefit of general hospital care than the general practitioners did. The nurses, on the other hand, were more confident in community hospital care. Other studies have demonstrated that specialists have a tendency to over-estimate the effect of their own specialty [20,26]. However, several studies in Norway, the Netherlands and UK confirm that appropriate care can be given at an intermediate level [27]; at community hospitals or at general practitioners hospitals [28-30]. We believe that this disagreement between professionals as to the benefit of a general hospital admission may be one of the greater challenges for the understanding of professional collaboration. There has to be a much better dialog between physicians at primary and secondary level to establish a consensus as to the definition of proper care, and what it entails. This may prevent unnecessary referrals to general hospitals and ensure appropriate follow-up care for patients after discharge from general hospitals.

Physicians' letters of poor quality are probably one of several factors contributing to inappropriate care. Without correct information about the patients' ADL and normal medical status, general hospital physicians have to deal with each disease as an isolated medical problem without any possibility of seeing the consequences of the present disease in the patient's daily social context. This in turn may result in discharge letters being written mostly from a general hospital point of view without necessarily addressing the problems that caused the referral in the first place.

This study, along with other similar studies [3-16], demonstrates the importance of establishing better systems for exchanging patient information between primary and secondary level. We also believe that it will be necessary, in the future, for health professionals to reach a consensus as to a definition of what is necessary information and appropriate care at primary and a secondary level. Today there would appear to be uncertainties between the health care levels about duties, responsibilities and possibilities of the care that can be provided by general hospitals or by primary care.

## Conclusion

In this study from one general hospital the quality of vital medical information between the health care levels and between physicians in order to provide appropriate care for older patients was insufficient and might represent potential health hazards for older patients. It is necessary to establish a better common consensus between health professionals as to the content and the form of professional communication between the care providers at primary and secondary level.

## Competing interests

The author(s) declare that they have no competing interests.

## Authors' contributions

HG and RJ developed the idea of, and the design of, the study together. HG was the project coordinator and mediator in the panels, performed the statistical analysis, interpreting the data and drafted the manuscript. RJ helped with the statistical analyses, interpreting the data and the drafting of the manuscript. Both authors have read and approved the final manuscript.

## Pre-publication history

The pre-publication history for this paper can be accessed here:


